# The Professional Life of London: How It Is Reacting to the War

**Published:** 1915-02-06

**Authors:** 


### THE PROFESSIONAL LIFE OF LONDON.

### How it is Reacting to the War.

The London medical world has a full share of the anxieties arising from the war. With few exceptions incomes have fallen, in some cases considerably, while expenses tend to move upwards rather than downwards. The general practitioner is probably doing as much work as ever, but the payment of his Christmas accounts tends to halt on the way. It is men in the Harley Street district who are the chief sufferers. To some extent the consulting physician or surgeon is a luxury, and in war-time a certain percentage of patients find they can do without him. Especially is this true of those engaged in the more specialised branches of practice. A little deafness or a nasal spur is not beyond human endurance, and at a push the present pair of spectacles can be made to carry on a little longer. Then, though London seems much the same, it is not really the same, for it has lost a large proportion of its visitors from the provinces, the Colonies, and the United States. Some proportion of these in ordinary times take the opportunity, when in town, to obtain an authoritative medical opinion, and so find their way to one or other of the professional celebrities who cluster in such numbers in the neighbourhood of Cavendish Square. Just now this current is a very feeble one, and it will hardly grow stronger until peace arrives. Reduced incomes form a hard experience, especially when the flag must always be kept flying. To resign the motor-car for the "Twopenny Tube" is a descent which for many reasons is out of the question. Let us hope that ere long prosperity may return to these high professional quarters. It would be a dreadful thing were the public to learn as a permanent conclusion that it is often possible to get on fairly well even without such distinguished assistance.

The medical societies, as well as individuals, are also feeling the prejudicial influence of the war. Meetings are much fewer, as programmes cannot be filled even by the most energetic secretaries. Many of the younger hospital men are involved in military duties either at home or at the Front, and others, as a consequence, have an increased burden of duties. There is thus neither time nor inclination for the collection of cases or the construction of papers, and the societies consequently suffer. Possibly the cynical will say the loss is not greatly to be regretted, as the multiplication of transactions, of reports, of journals, and indeed of medical literature generally, has of recent years been enormous. There have been interesting and useful discussions on the surgery of the war, and the record of many of these will certainly have permanent value.

The question of medical prisoners of war has been revived by the release by the Germans of three officers of the R.A.M.C., who have been in captivity since September, and who certainly relate some very unhappy experiences. The rule of the Geneva Convention is that medical officers who fall into the hands of the enemy shall not be treated as prisoners of war, but shall carry on their duties under the direction of the enemy, and when their assistance is no longer indispensable they shall be sent back to their army or their country. This rule has certainly not been followed in the present war, and each side seems to blame the other for the neglect of it, and to excuse its own inaction on account of this neglect. No official statement has been made of the number of German doctors detained in this country, and presumably some are kept for the medical care of prisoners and others. But in consequence of the demands of the armies there is a short supply of doctors for the civil population in all the fighting countries, and in these circumstances the detention of doctors as prisoners of war is nothing less than a crime, as well as a breach of the Geneva Convention. A recent letter in the *Times* from Sir John Byers supports the plea advanced in The Hospital on November 7, that all medical officers should be released at the earliest possible moment. The nation which sets the good example will have the greater honour.

Complaints continue to be heard on all hands of the difficulty of obtaining resident house physicians and house surgeons for the hospitals. Vacancies are advertised again and again, and often without gaining a single reply. Increases of salary even to the extent of 50 to 100 per cent. are in vain. This has led to very bright expectations in the Women's School, and there is no doubt that the young ladies have just now opportunities such as they never had before.

As we note in "Round the World," among the recent departures for the Front in Mr. James Berry, who has gone out in charge of one of the Serbian hospitals. Everyone knows his excellent work on the surgery of goitre, and his many friends will watch his new departure with interest, and will heartily wish him a safe return.

In medical circles the opponents of anti-typhoid inoculation gain little attention. They are discredited by their "past" and by their exaggerations. Most people agree that so far as the present emergency is concerned a sufficient word has been spoken by Sir William Osler in his letter to the *Times*. Appeals and exhortations by others who have little personal knowledge of the subject are certainly superfluous, and may be harmful.

The new Pharmacopœia has been received very calmly in medical circles. The fact is that its issue has little effect on the present generation of practitioners, whose prescribing habits are fixed and are not to be upset by the edict of the General Medical Council. Some few changes must needs be noted, as, for example, the alterations in some of the more active tinctures, but these are felt as occasions for grumbling rather than gratitude. Pharmacists and students are more concerned than the medical practitioner, and perhaps after all the issue of the new volume is not so earth-shaking an event as its authors imagined it would be.

